# Acupuncture and Related Therapies for Symptom Management in Palliative Cancer Care: Systematic Review and Meta-Analysis: Erratum

**DOI:** 10.1097/01.md.0000484109.00290.e6

**Published:** 2016-05-20

In the article “Acupuncture and Related Therapies for Symptom Management in Palliative Cancer Care: Systematic Review and Meta-Analysis”,^1^ which appeared in Volume 95, Issue 9 of *Medicine*, Dr. Sit's name was originally spelled incorrectly, and Dr. Ng's degree was listed incorrectly. The article has since been corrected online.
